# Extreme Environmental Stress-Induced Biological Responses in the Planarian

**DOI:** 10.1155/2020/7164230

**Published:** 2020-06-11

**Authors:** Zhonghong Cao, Hongjin Liu, Bosheng Zhao, Qiuxiang Pang, Xiufang Zhang

**Affiliations:** School of Life Sciences, Shandong University of Technology, 266 Xincun Western Road, Zibo 255049, China

## Abstract

Planarians are bilaterally symmetric metazoans of the phylum Platyhelminthes. They have well-defined anteroposterior and dorsoventral axes and have a highly structured true brain which consists of all neural cell types and neuropeptides found in a vertebrate. Planarian flatworms are famous for their strong regenerative ability; they can easily regenerate any part of the body including the complete neoformation of a functional brain within a few days and can survive a series of extreme environmental stress. Nowadays, they are an emerging model system in the field of developmental, regenerative, and stem cell biology and have offered lots of helpful information for these realms. In this review, we will summarize the response of planarians to some typical environmental stress and hope to shed light on basic mechanisms of how organisms interact with extreme environmental stress and survive it, such as altered gravity, temperature, and oxygen, and this information will help researchers improve the design in future studies.

## 1. Introduction

Planarians are bilaterally symmetric metazoans of the phylum Platyhelminthes. They have well-defined anteroposterior and dorsoventral axes and have an anterior cephalic region which contains the brain and a pair of eyespots, a central region which includes the pharynx and the mouth, and a posterior tail region. Despite their relatively simple morphology, planarians have a highly structured central nervous system (CNS) and feature a true brain which consists of all neural cell types and neuropeptides found in vertebrates [1–3]. They have roughly 30% adult stem cells [4, 5] and possess an extensive potential of regeneration. Planarians are one of the few animal species that can easily regenerate their head including the complete neoformation of a functional brain within seven days after decapitation [6–9]. Moreover, planarians share more genes with vertebrates compared with other popular model organisms such as *Drosophila melanogaster* or *Caenorhabditis elegans* [10]. All of these make planarians a reliable and popular model in the field of developmental, regenerative, and stem cell biology.

Exploring and living somewhere beyond the Earth are always two of the dreams of humans. It means that humans had successfully walked the first step to explore the space when Soviet cosmonaut Yuri Alekseyevich Gagarin had finished his journey of 89 minutes of orbiting the planet in his space capsule, on April 12, 1961. Then, more and more astronauts successfully finished their spaceflight, and their dwell time in space also is longer and longer. But the bad news is that astronauts on a long-term mission have problems upon returning to Earth, such as bone density loss; muscle atrophy; cardiovascular and hematic changes; metabolic, endocrine, and sleep disturbances; and rapid senescence [11, 12]. These actual and potential physical effects on the body mainly come from extreme environmental stress outside the Earth, such as altered gravity, temperature, and oxygen. Then, if we want to provide a habitat that can keep organisms in it living, it is necessary to understand what the biological responses of extreme environmental stress induce. We ethically cannot directly test the impact of environmental stress on humans. We need to use appropriate animal models to learn the adaptation of mammals about environmental stress.

Planarians not only can easily regenerate the lost parts but also can survive a series of altered environment. These make the planarian an ideal model organism, help us to understand how extreme environmental stress impacted the biological responses of organisms, and help us to find out the conserved molecules and mechanisms that support organisms to survive extreme environmental stress. There are reports that environmental stress can impact the structure of cells, intercellular communication, regeneration, embryonic development, and even immunological responses [13–18]. In this review, we will summarize the impact of some typical environmental stress for the planarian and hope to shed light on basic mechanisms of how organisms interact with extreme environmental stress and survive it, such as altered gravity, temperature, and oxygen. And this information will help researchers understand the basic mechanisms and improve the design in future studies.

## 2. Physiological Effects of Altered Gravity and Magnetic Field

Later missions showed that space travel could be tolerated by humans, but microgravity will trigger lots of physiological responses and even some pathological reactions. If we want to keep organisms healthy and happy in altered gravity, we need to use appropriate animal models to learn the effects of altered gravity. Planarian flatworms possess remarkable regeneration ability and share more genes with vertebrates than other popular model organisms such as *Drosophila melanogaster* or *Caenorhabditis elegans* [10], so it can minimize background interference, and becomes an ideal model to learn the effects of altered gravity. This part will summarize the most relevant data from exposure of planarian to altered gravity and magnetic levels obtained through ground-based facilities or board spaceflights, sounding rockets, satellites, or space stations. We will discuss the different effects of altered gravity on planarian, including the impacts on regeneration ability, embryonic development, phototaxis response, moving behavior, and transcriptomic information.

### 2.1. The Regeneration and Fission Ability

In the study of Gorgiladze et al., they cut 60 freshwater planarian *Girardia tigrina* 12 to 14 h before the spacecraft starts from the Baikonur launching site. In their experiment, they cut off the planarians before and after the pharynx and collected the different fragments into 20 ml polyethylene vials which are filled with freshwater, respectively. The air temperature of the RS ISS service module ranged from 19 to 21°C, as determined by telemetry. After 10 days of journey, they found that all the amputated body parts regenerated the lost parts, and the morphometric parameters of regenerated fragments were not different from those of the control fragments. And yet, the regenerated planarians were smaller than the original “maternal” planarians until the 18th to 20th day after dissection [19].

**Figure 1:**
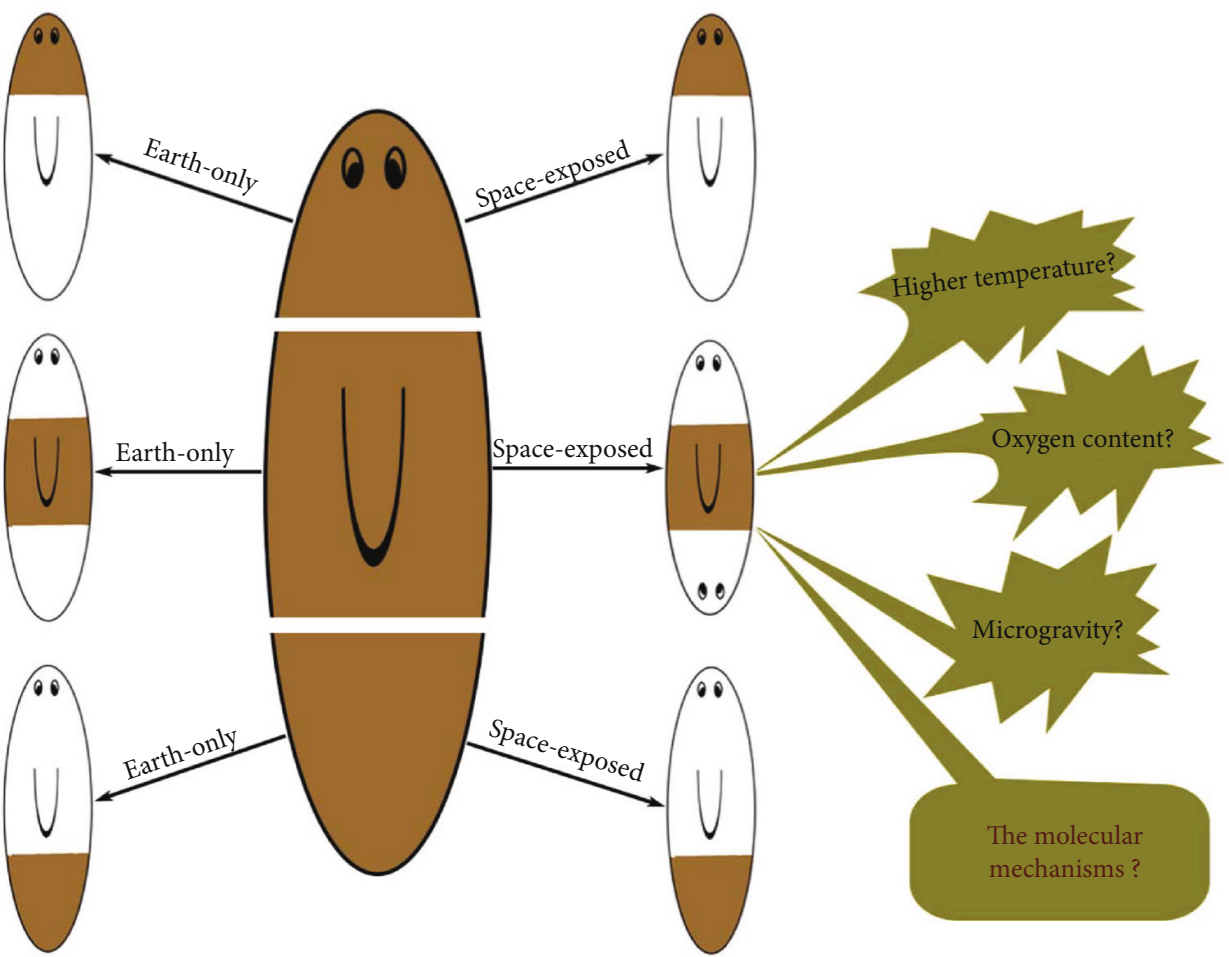
The effects of changed gravity on regenerated tissues in planarian. The left panel indicates that Earth-only planarian fragments regenerated the lost part. The right panel indicates that space-exposed head and tail fragments regenerated the lost parts, but the truncated middle planarian regenerated two heads. The immediate cause and molecular mechanisms are not well known for this phenomenon. Brown marks the original tissue, and white marks the regenerated parts.

Whole-mount and amputated fragments of *Dugesia japonica* planarian had been collected into sealed 50 ml tubes with 50%/50% air/water, then had been sent to the ISS for one month. Results showed that only the whole worms are divided spontaneously which had been sent into space; the fission rate changed from 1.3 to 1.75. They did not find a fission phenomenon in other samples. Yet, the authors cautioned that the worms in space unavoidably experienced somewhat higher temperatures at some time periods, so we should keep admonishing for this result. After returned two months later, the number of worms that had gone to space was slightly less than the worms that were maintained on Earth [20]. For the amputated worms, the size is similar between the two groups after the space journey. And after two months of culture, the worms exposed to space grew more slowly than the Earth-only controls. The most striking phenomenon is that they found that one of the 15 pharynx fragments from space had regenerated two heads (see Figure 1), and after amputating the two heads, the headless middle fragment regenerated into a double-headed phenotype [20]. But we need to note that pharynx fragments left on Earth did not survive the duration of the mission in this work. Levin et al. thought that people should be cautious about this result; maybe it is not really induced by microgravity [21].

Teresa et al. performed their experiment in the European Space Research and Technology Centre, Noordwijk, The Netherlands. In their work, they researched the impact of simulated microgravity on the regeneration of *Schmidtea mediterranea*. They get simulated microgravity by means of the random positioning machine (RPM) set at a speed of 60°/s and 10°/s, and their results demonstrate that RPM 60°/s led to the death of trunk planarians, whereas planarians loaded into the 10°/s RPM machine appeared normally regenerated (have the normal eyes and the normal CNS and have similar mitotic activity). Moreover, they found that all planarians live and correctly regenerated the corresponding lost parts on day 5, which indicates that planarians do not die soon when sensing the effects of the 60°/s RPM but after having regenerated the main structures. They hypothesized that there are rheoreceptors in the head of planarians, and when they regenerated the whole head, they can sense the water currents which are produced in the 60°/s RPM and induce death [16]. It is necessary to analyze the effect of 60°/s RPM rotation for intact animals and definitely corroborate this hypothesis. These results demonstrate that it is not the simulated microgravity but the specific setting of rotation of the 60°/s RPM that induced the death of planarians.

They also examined the impact of hypergravity on regeneration of *Schmidtea mediterranea* and simulated hypergravity 3g, 4g, and 8g by a large diameter centrifuge (LDC). Under 3g and 4g hypergravity, planarians can regenerate missing tissues, but the proliferation rate was decreased. Under 8g hypergravity, only the larger trunk planarian fragments can regenerate the lost part, and the small planarian fragments cannot successfully regenerate the corresponding part. Although the molecular reason for this effect has not been found, the decreased proliferative rates suggest that gravity could affect the actin cytoskeleton and the assembly of microtubules, just as it happens in other organisms [22–24]. Meanwhile, they found that changed gravity had affected the fission rate of planarians, and the number of smaller fragments was significantly higher in LDC 4g planarians compared with the rest of the groups.

The study about 3–6 d old cocoons of *Schmidtea polychroa* showed that all cocoons in RPM 60°/s or LDC 3g conditions were taken out on the corresponding day. Immunohistochemical results of *α*-tubulin showed that all juveniles had a proper morphology and normal organization of the CNS and pharynx. And all animals have a similar mitotic activity. These results demonstrate that planarians can develop properly in altered gravity conditions, at least in the specific settings used in this experiment. And it called for further analysis for a more complete understanding if juveniles can survive longer times in the RPM 60°/s conditions [16].

In the research of Lu et al., they changed the gravity conditions through a large gradient high magnetic field (LG-HMF) and observed regeneration of planarians at three gravity levels (ug, 1g, and 2g). Their results demonstrate that all of the planarians normally regenerated their heads and the regeneration rate of the four groups is similar. These results indicate that planarians can correctly regenerate their heads at different gravity environments [25].

Sousa et al. in the European Space Research and Technology Centre (Noordwijk, The Netherlands) through the random positioning machine (RPM) simulated microgravity (the machine was set to a real random mode and random direction with a maximum speed of 10°/s), and the large diameter centrifuge (LDC) generated hypergravity (8g). They put intact *Schmidtea mediterranea* planarians in the RPM and LDC at day 0; after 1 day, the planarians were amputated at pre- and postpharynx levels, and they collected trunk fragments and reloaded in the same devices. They found that all the planarian trunks exposed to s-ug or 8g correctly regenerated the lost parts [26]. There are results showing that microgravity can induce developmental retardation and cell apoptosis of mouse embryos [27], and hypergravity conditions do not affect the normal development and actin filament structures of mouse embryos [28]. But microgravity can prevent terminal differentiation of embryonic stem cells [29, 30].

There are researchers that treat decapitated planarians with weak nonuniform magnetic fields (RMFs) and found RMFs eliminated the formation of edema and blastema, which through upregulating the expression of EGR4, Netrin 2, NSE, and NPY accelerates nerve cell proliferation and function recovery [31–33]. The weak magnetic field stimulates the fission frequency of the Planarian *Dugesia* (*Girardia*) *tigrina* [32]. Alanna et al. recently found that weak magnetic fields change the accumulation of reactive oxygen species (ROS), ERK cascade, and the expression of heat shock protein 70 (Hsp70) to regulate the proliferation and differentiation of stem cells [34, 35]. The planarian will die suddenly when the geomagnetic activity is over K6 [36].

### 2.2. Behavior

In the experiment of Gorgiladze, the regenerated planarians have normal food behavior and locomotor activity; they can freely glide or alternate contraction and straightening of the body on the bottom of the flask or on the water surface [19]. Morokuma et al. had sent 5 tubes with different numbers of whole worms to the ISS and found that only the sample which contains 10 whole worms that had been launched into space showed immediate unusual behavior. They curled up ventrally and are somewhat paralyzed and immobile, when they had been introduced into fresh Poland spring water. They all regained normal behavior after 2 hours. And water shock was not seen in the other samples. These results indicate that microgravity can yield different effects according to different microenvironments of culture systems. After 20 months of return to Earth, the two groups of worms showed a comparable motion rate under the stimulation of red and blue light, and the control worms spent more time in the dark compared to the space-traveled worms [20].

Lu et al. found that the planarians which regenerated heads in different gravity conditions showed similar photonegative response. During the photonegative test, most of the regenerated planarians could reach the target quadrant in 90 s, and the average times spent in the target quadrants did not significantly differ. These results showed that LG-HMF-generated microgravity and hypergravity did not affect the reestablishment of photonegative ability. But the photonegative response time of the planarians which regenerated under LG-HMF conditions was slightly suppressed, and the authors thought it was mainly due to the difference of the locomotor system instead of the reconstructed head [25]. They took the traditional planarian locomotor velocity (pLMV) assay and automated center-of-mass (COM) tracking approach and image analysis to analyze the locomotor behaviors, and the results showed that Group ug/12T has a significantly decreased locomotor function compared to the other three groups during the 8 min test. And the righting time of the simulated microgravity group also showed significantly increased compared to the other groups, but the planarians could eventually sense the reverse direction and complete the correction of the body, indicating that the function of the nervous system was normal. Histologic section staining and immunohistochemistry results showed that the circular muscle of planarians regenerated in simulated microgravity was weakened compared with the other planarian groups, and the fluorescence thickness of the epithelial cilia are significantly decreased. The authors thought that differences in locomotion velocity and righting behavior come from frail muscle [25]. There is research showing that planarian that has been exposed to 16G intensity static magnetic fields for one day significantly improved the velocity of movement [37].

### 2.3. Transcriptomic

In the experiment of Sousa et al., they put intact *Schmidtea mediterranea* planarians in the RPM and LDC at day 0. After 1 day, the planarians were amputated at pre- and postpharynx levels, and they collected trunk fragments and reloaded in the same devices. Five and 12 days after amputation, they collected all kinds of samples, respectively, and analyzed the transcriptome of each sample. The principal component analysis (PCA) showed that the same time samples were clustered together and the same gravity condition samples were also clustered together. They found that after 12 dR (13 days of s-ug or 8g exposure), several genes were deferentially expressed in exposed animals compared to their corresponding controls. The number of differentially expressed genes was much higher in animals regenerating in s-ug conditions than in animals regenerating at 8 g, and there is a much higher number of deregulated genes (720 versus 77) [26].

Regarding the specific deregulated genes at s-ug, they found the downregulation of cytoskeleton and matrix genes—such as *collagen-a-1*, *piwi* genes, and the upregulation of genes which are involved in ribosome biogenesis. What makes them confused is that although supporting higher mechanical forces require strengthening the cell cytoskeleton to maintain the shape and function, they did not find significant alteration of cytoskeleton or matrix proteins in planarians regenerating at 8g conditions. Their results indicate that altered gravity conditions can severely affect genetic transcription, and these alterations potentiate molecular disorders which could promote the development of multiple diseases such as cancer [26].

### 2.4. The Others

In the study of Morokuma et al., the microbiome profiles of those culture-based are significantly different. In space-exposed worms, the number of colonies of *Variovorax*, *Herminiimonas*, and the unknown *Comamonadaceae* decreased and the number of *Chryseobacterium* colonies significantly increased. Their results indicate that space travel can change bacterial community composition of *D. japonica*, and this difference can exist for a few years. And they analyzed the samples of the water of the space-exposed worms and the Earth-only with liquid chromatography-mass spectrometry (LC-MS); the results revealed that both samples contained a large number of small organic molecules/metabolites. The total ion chromatograms of the two samples in the positive ion mode were quite different, and many of them correspond to long-chain fatty acids or monohydroxylated/dihydroxylated long-chain fatty acids [20].

## 3. Physiological Effects of Temperature and Oxygen

Temperature and oxygen are the other important factors of the environment, which can affect, regulate, and control lots of biological and pathological processes of organisms [6, 38–40]. For organisms, sensing the temperature and oxygen of the environment is very important for them to adjust behavioral strategy and escape injury. There are researchers reporting that nutrition and temperature can impact the oxygen consumption and metabolic status and impact the process of development, regeneration, injury, and escape from noxious stimulation, etc. [41–44]. And environmental stress can, through a conserved pathway, impact the biological process from planarian to human. In this session, we will discuss the impact of temperature and oxygen on planarian.

### 3.1. The Regeneration and Fission Ability

People found that the ROS production takes part in the regeneration in *zebrafish* and *Xenopus* [45, 46]. But the limited regeneration ability of these organisms restricted researchers that deeply explore the function and impact of ROS in regeneration. Planarians are famous for their amazing regeneration ability; they can easily regenerate any parts of the body and include a functional head, and the new head can even have the memory of the former brain. These characters make these worms to be an ideal model.

Every live organisms need energy to maintain the function of cells; hence, every living cell needs to consume oxygen to keep the balance of metabolism. There are researches reporting that small planarians have a higher oxygen consumption rate, and injured planarians have a lower oxygen consumption rate, but injured worms have increased glycolysis during the process of regeneration [47]. Pirotte et al. researched the impact of ROS on planarian regeneration with *Schmidtea mediterranea.* They amputate the planarian into three fragments at pre- and postpharynx levels and research the impact of ROS to different part regeneration. They found that ROS burst just in a few minutes after amputation, and the production of ROS is independent of the orientation of the wound site, but it induced signals to regulate the regeneration process which appears at least after 24 h from amputation. Inhibition of the production of ROS leads to failure to regenerate the lost parts of all three fragments, and they found that reduced ROS restricted the regeneration of cephalic ganglia and the ectopic neuronal cells. And they found that disturbing the production of ROS did not affect the stem cell proliferation but restricted the neoblast differentiation into the required cell types of regeneration [48]. In addition, there are works that suggest that increased ROS do not accelerate the aging of mice and some long life-span mammals have a higher level of ROS and oxidative damage [49, 50]. Literatures showed that human protein has Met and Cyst residues, and these residues through trapping oxygen atoms prevent ROS-induced neuronal cell death [51, 52]. Tsushima et al. found that the protein of DJ-1 is conversed from human to planarian, especially the important residues for function execution; they knock down the DJ-1 gene in vivo through RNAi, and the results showed that planarian DJ-1 has antioxidant and neuroprotective functions; it indicates that planarian can be a reliable model for study oxidative stress-introduced disorders and offer the chance to explore the mechanisms [53].

There is literature reporting intact planarian preference to move to the cooler region, and even the amputated head fragment moved to the cold field [54]. It means that the head region can sense and responds to environment temperature in planarian and showed that DjTRPMa-expressing neurons sense the temperature and transduce signals to serotonergic neurons of the brain; then, serotonergic neurons exhibit thermotactic behavior. Ding et al. reported that *Dugesia japonica* planarian showed different regeneration speeds at different temperatures (15°C, 20°C, and 25°C); lower temperature decreased the regeneration speed [55]. The *Schmidtea mediterranea* trunk fragment can completely regenerate the head and tail at five days after amputation when cultured at 26°C and 28°C and shortened to two days compared with the planarian cultured at 19°C. And the eyes appeared from three days postamputation when cultured at 26°C and 28°C, but the control worms regenerated eyes at five days [56].

According to literatures showing that the fission of planarian flatworms correlates with the length and area size of worms [57], the fission frequency increased with the body size; when the body length is shorter than 4-5 mm, they cannot fission again [58]. Subsequent researchers reported that environmental stress can impact the process, such as increased temperature would decrease the fission length and increase the frequency of fission [59]. Hammoudi et al. showed that the spontaneous fission frequency multiplied significantly at 26°C and 28°C than at 19°C [56]. In addition, there have been reports that before the fission event, there was an increased proliferation of neoblast just like after amputation [60], and activating the mitotic functions through RNAi of DjP2X-A can induce higher fission frequency [61].

### 3.2. Behavior

Planarians can normally live and behave from 15°C to 25°C, the locomotor activity has been strongly suppressed below 10°C, the worms will lose their motility between 5 and 10°C, and high temperature almost did not affect the mobility of planarians, but they will die in 1 hour when the temperature is above 30°C [54, 56]. Hammoudi et al. reported that slowly increasing the temperature of water can elongate the live times of planarians, but they cannot survive more than 20 days when the temperature is over 30°C [56]. From 7°C to 12°C, the body of planarian has some contraction, movement is slow, and the velocity is not stable; when the temperature is between 12°C and 21°C, the velocity gradually increased to its maxim value and the body stretched along the anterior-posterior axis which probably extended 25 per cent compared with that at 10°C. Above 21°C, the locomotor rate becomes not constant again and the speed is no more than that at 21°C. When the temperature increased to 30°C, the worms become motionless [62].

Ding et al. showed that suitable living temperatures can accelerate the toxic effect of Fe^3+^. They observed the toxic effect of Fe^3+^ for planarian at three different temperatures 15°C, 20°C, and 25°C and found that the death speed increased at 20°C and showed the lowest death speed at 15°C [55]. Normally, dorsal epidermis of planarians has excretory pores, hair cells, and rhabdites and can secrete droplets and generate mucus. The structure of epidermis has been damaged when the temperature increased over 33°C for *Girardia tigrina* and 37°C for *Girardia* sp. There were fewer rhabdites and fewer and disorganized secretory droplets [63], which form the mucus to help planarians to respond and escape stress [64, 65].

Higher temperatures did not impact the feeding behavior of planarians from 19°C to 28°C [56]; the ability to eliminate bacteria of planarians at different temperatures changed. After infection with 10^9^ CFU of *S. aureus* for three hours, the worms need six days to eliminate the bacteria at 19°C and just need three days when they had been cultured at 28°C. It means that planarians have exacerbated antibacterial capabilities with the increase of temperature from 19°C to 28°C [56]. The eye action potential (OP) that evoked by a light flash in the planarian changed with the temperature. When the temperature increased from 15°C to 23°C, the amplitude increased and the latency and peak delay decreased; as the temperature is greater than 30°C, the amplitude decreased and latency and peak delay continued to decrease until 42°C. These changes can be reversible when the temperature is lower than 30°C [66].

Ectothermic organisms respond to altered temperatures through adjusting their biochemical process but not through changing their body temperature [67]. One of the important strategies is through changing the mitochondrial oxidative phosphorylation (OXPHOS) pathways to survive, which is critical to provide energy for the eukaryotic cells. Hence, OXPHOS is important in balancing the process of metabolism and the generation of reactive oxygen species (ROS). And moderate ROS is necessary for lots of important biological processes, but overproduced ROS can damage DNA, lipids, and proteins [68]. It means temperature changes OXPHOS to modify the production of ROS and then affect the signals of cells [69, 70]. Animals can respond to temperature through two steps, one is the phosphorylation system and the other is the complex I of the NADH pathway [71–75]. After 4 weeks of acclimation under low temperature, planarians can effectively increase the capacity of these related proteins [75].

### 3.3. The Molecular Response

There is literature showing that the conserved ion channel TRPA1 exhibit important function to feel noxious heat and start the protective behavior [76], but Arenas et al. showed that TRAP1 cannot be directly activated by heat; they found that heat induces the production of ROS and then through RTPA1 regulates the escape process in *Schmidtea mediterranea* planarian [77]. Disturbing the regeneration of ROS or the expression of RTPA1 can lead to failure of avoiding noxious heat.

When the temperature increased to 25°C, the expression of DjHsp90 protein upregulated approximately 2-fold compared with that cultured at 18°C. When the temperature increased to 32°C, the DjHsp90 protein level decreased lower than normally (18°C) cultured planarians, but the level of *Djhsp90* mRNA is still higher at 32°C. It means that high temperature firstly restricted the process of posttranscription events [78]. Immunohistochemistry showed that the *Djhsp90*-positive cells distributed in parenchymal tissue from head to tail, just under the epidermis cells, indicating that they are suitable to sense and respond to the external environmental signals [78]. When temperature changed (increase or decrease), head-expressed *DjSpsb* mRNA will increase in *Dugesia japonica* [79]. The levels of putrescine and spermidine are also temporarily increased after heat shock or cold shock, which is essential for planarian to recover from damage [80].

## 4. The Controversial Issues and Future Directions

People created different experiments to explore the impact of extreme environmental stress on planarians and had obtained lots of valuable information (Table 1). But there are still some controversial issues that need to be noted and improved in future research.

### 4.1. Planarian Species and Planarian Density

In different researches, people used different planarian species, such as *Schmidtea mediterranea*, *Dugesia tigrina*, and *Dugesia japonica*. Jason et al. had reported that even though different species share similar anatomy and mode of reproduction, they find that each species had acquired its own distinct strategy for optimizing its reproductive success [81]. The other question is the density of planarian. In present reports, the researchers placed different numbers of planarian in a single tube and resulted in different densities of worms in each experiment. And the density of worms can impact the fission and the expression of some molecules. This makes it complicated to directly compare the results. We need to take into account the impact of planarian species and planarian density if we want to get more reliable and to directly compare data.

### 4.2. The Methods and Strategies to Change Environmental Stress

Just as mentioned above, researchers use different strategies and methods to change the environment station. And these differences can lead to inconsistent results, such as the fact that the same microgravity conditions in RPM 60°/s can lead to the death of regenerated planarians but not in 10°/s; slowly increasing the temperature to 30°C makes planarian live longer times. Further analysis showed that the rheoreceptors of the intact head induced the death of planarians in RPM 60°/s but not the microgravity. When analyzing the effects of given environmental stress, we need to be cautious to the results and carefully consider the method and strategy to change the stress condition.

### 4.3. The Crosstalk of Different Environmental Stress

**Figure 2:**
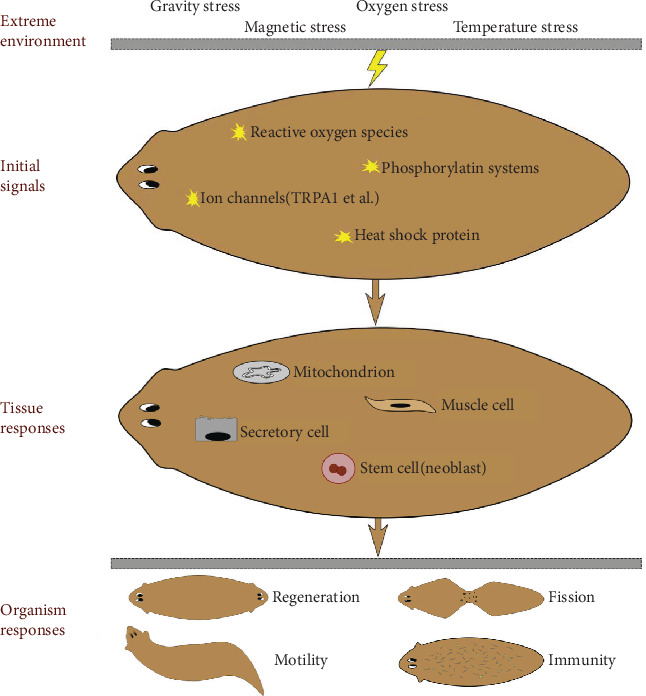
The effects of extreme environmental stress on planarians. Different kinds of extreme environmental factors irritate planarian; organisms sense it and produce the initial signal molecules (yellow star), then recruit more respond factors to join the war and change the metabolism status of different cells and organelles; at last, the organism showed changed regeneration ability, fission frequency, mobility, and immunity.

Existing research showed that microgravity did not induce failure of regeneration, but the regeneration becomes slow. And microgravity increased the spontaneous fission frequency and changed the microbiome profiles of planarian. They curled up and are immobile in spring water when they returned to Earth from space, then slowly recover to normal mobility after two hours. Researchers did not find upregulated genes of cytoskeleton or matrix related to hypergravity which has been supposed to maintain shape and strength of the cytoskeleton of cells. Temperature and oxygen are important environmental factors; oxygen is necessary for almost all animals. Research showed that temperature can impact the oxygen consumption and metabolic status, impact regeneration capacity and fission frequency, and even regulated the capacity to eliminate bacteria. Just as Figure 2 shows that different environmental factors irritate organisms, organisms sense it and produce the initial signal molecules, then recruit more response factors to join the war and change the metabolism status, protein expression levels, cell structures, and so on; at last, the organism exhibits the environmental stress effects, such as changed regeneration ability, fission frequency, mobility, and immunity. Different environmental factors can generate cross effects; there are researches showing that late loading and early retrieval can increase the success of life science experiments in space [82, 83]. Long time cold storage will increase the death frequency and induce fail of regeneration [84].

## 5. Conclusion

The relatively simple planarian offered a unique opportunity to study the physiological and behavioral process and to investigate the mechanisms underlying different environmental stress in whole animals. It is more helpful to understand the overall response of the organisms to facing different environmental stress than specific tissues. These could provide us with important insights about basic molecular processes occurring in a wide range of vertebrates, including humans. But present researches analyzed the morphological and physiological changes more than molecular mechanisms, and some researches even get conflicting results for the same stress condition. These results call for further experiments to carefully consider the cross effects of different environmental stress and deeply detect the molecular mechanisms. It is still an exciting era for researchers.

## Figures and Tables

**Table 1:** Some major advances of the effect of extreme environmental stress on planarians.

Stress conditions	The main impact on planarian	References
The effects of gravity and magnetic field		
Microgravity (space)	All the amputated body parts regenerated the lost fragments.	Gorgiladze [19]
Microgravity (space)	The whole worms had spontaneous fission. The pharynx fragment had regenerated two heads and grew more slowly. Some whole worms showed immediate unusual behavior. The microbiome profiles had changed.	Morokuma et al. [20]
Microgravity (RPM 60°/s)	The trunk planarians had died.	Adell et al. [16]
Microgravity (RPM 10°/s)	The trunk planarians appeared normally regenerated.	
Hypergravity (LDC 3g, 4g)	The amputated body parts regenerated the missing tissues; the proliferation rate was decreased.	
Hypergravity (LDC 8g)	Only the larger trunk planarian fragments can regenerate the lost part.	
Microgravity (LG-HMF ug)	The amputated body parts normally regenerated their heads. The worms have a significantly decreased locomotor function.	Lu et al. [25]
Hypergravity (LG-HMF 2g)	The amputated body parts normally regenerated their heads.	
Microgravity (RPM 10°/s)	The body parts properly regenerated head. Cytoskeleton and matrix genes had been downregulated.	Sousa et al. [26]
Hypergravity (LDC 8g)	The body parts properly regenerated head. Microtubules, cell communication, and cell cycle genes had been downregulated.	
Weak magnetic field	The nerve cell proliferation has been accelerated. The regeneration speed increased. The frequency of spontaneous increased. The velocity of movement improved.	Novikov et al. [32], Gang et al. [33]. Gang and Persinger [37]
Intense magnetic field	The planarian will suddenly die when it is over 6K.	Murugan et al. [36]

The effects of oxygen and temperature		
Inhibit ROS	Planarian fragments fail to regenerate the lost parts. The regeneration of cephalic ganglia and ectopic neuronal cells had been restricted. Neoblast differentiation has been restricted.	Pirotte et al. [48]
Increase ROS	Induce damage of DNA, lipids, and proteins.	Finkel [68]
Lower temperature (15°C)	The regeneration speed decreased. The spontaneous fission frequency increased. The movement is slow, and the velocity is not stable. The toxic effect of Fe^3+^ has been decreased.	Ding et al. [55], Hammoudi et al. [56], Herath and Lobo [59], Cole [62]
Higher temperature (25-33°C)	The regeneration speed increased. The movement is slow, and the velocity is not stable. The secretory function has been restricted. The capability to eliminate balance increased.	Ding et al. [55], Hammoudi et al. [56], Cole [62], Oliveira et al. [63]

## References

[1] Collins J. J., Hou X., Romanova E. V. (2010). Genome-wide analyses reveal a role for peptide hormones in planarian germline development. *PLoS Biology*.

[2] Mineta K., Nakazawa M., Cebria F., Ikeo K., Agata K., Gojobori T. (2003). Origin and evolutionary process of the CNS elucidated by comparative genomics analysis of planarian ESTs. *Proceedings of the National Academy of Sciences of the United States of America*.

[3] Fraguas S., Barberán S., Ibarra B., Stöger L., Cebri F. (2012). Regeneration of neuronal cell types in Schmidtea mediterranea: an immunohistochemical and expression study. *The International Journal of Developmental Biology*.

[4] Sarnat H. B., Netsky M. G. (1985). The brain of the planarian as the ancestor of the human brain. *The Canadian Journal of Neurological Sciences*.

[5] Agata K., Soejima Y., Kato K., Kobayashi C., Umesono Y., Watanabe K. (1998). Structure of the planarian central nervous system (CNS) revealed by neuronal cell markers. *Zoological Science*.

[6] Reddien P. W., Alvarado A. S. (2004). Fundamentals of planarian regeneration. *Annual Review of Cell and Developmental Biology*.

[7] Forsthoefel D. J., Newmark P. A. (2009). Emerging patterns in planarian regeneration. *Current Opinion in Genetics & Development*.

[8] Salo E., Abril J. F., Adell T. (2009). Planarian regeneration: achievements and future directions after 20 years of research. *The International Journal of Developmental Biology*.

[9] Brown D. D. R., Pearson B. J. (2017). A brain unfixed: unlimited neurogenesis and regeneration of the adult planarian nervous system. *Frontiers in Neuroscience*.

[10] Sanchez Alvarado A. (2006). Planarian regeneration: its end is its beginning. *Cell*.

[11] Buckey J. C. (2006). *Space Physiology. Oxford*.

[12] Demontis G. C., Germani M. M., Caiani E. G., Barravecchia I., Passino C., Angeloni D. (2017). Human pathophysiological adaptations to the space environment. *Frontiers in Physiology*.

[13] Sarkar D., Nagaya T., Koga K., Seo H. (1999). Culture in vector-averaged gravity environment in a clinostat results in detachment of osteoblastic ROS 17/2.8 cells. *Environ Med*.

[14] Uva B. M., Masini M. A., Sturla M. (2002). Clinorotation-induced weightlessness influences the cytoskeleton of glial cells in culture. *Brain Research*.

[15] Sundaresan A., Risin D., Pellis N. R. (2002). Loss of signal transduction and inhibition of lymphocyte locomotion in a ground-based model of microgravity. *In Vitro Cellular & Developmental Biology. Animal*.

[16] Adell T., Saló E., van Loon J. J. W. A., Auletta G. (2014). Planarians sense simulated microgravity and hypergravity. *BioMed Research International*.

[17] Lei X., Deng Z., Zhang H. (2014). Rotary suspension culture enhances mesendoderm differentiation of embryonic stem cells through modulation of Wnt/*β*-catenin pathway. *Stem Cell Reviews and Reports*.

[18] Lei X., Cao Y., Ma B. (2020). Development of mouse preimplantation embryos in space. *National Science Review*.

[19] Gorgiladze G. I. (2008). Regenerative capacity of the planarian Girardia tigrina and the snail Helix lucorum exposed to microgravity during an orbital flight on board the International Space Station. *Doklady Biological Sciences*.

[20] Morokuma J., Durant F., Williams K. B. (2017). Planarian regeneration in space: persistent anatomical, behavioral, and bacteriological changes induced by space travel. *Regeneration (Oxf)*.

[21] Levin M., Morokuma J., Finkelstein J. (2017). Space travel has effects on planarian regeneration that cannot be explained by a null hypothesis. *Regeneration (Oxf)*.

[22] Papaseit C., Pochon N., Tabony J. (2000). Microtubule self-organization is gravity-dependent. *Proceedings of the National Academy of Sciences of the United States of America*.

[23] Crawford-Young S. J. (2006). Effects of microgravity on cell cytoskeleton and embryogenesis. *The International Journal of Developmental Biology*.

[24] Vorselen D., Roos W. H., MacKintosh F. C., Wuite G. J. L., Loon J. J. W. A. (2014). The role of the cytoskeleton in sensing changes in gravity by nonspecialized cells. *The FASEB Journal*.

[25] Lu H.-M., Lu X.-L., Zhai J.-H. (2018). Effects of large gradient high magnetic field (LG-HMF) on the long-term culture of aquatic organisms: planarians example. *Bioelectromagnetics*.

[26] de Sousa N., Rodriguez-Esteban G., Colagè I. (2019). Transcriptomic analysis of planarians under simulated microgravity or 8 g demonstrates that alteration of gravity induces genomic and cellular alterations that could facilitate tumoral transformation. *International Journal of Molecular Sciences*.

[27] CAO Y., FAN X., SHEN Z., MA B., DUAN E. (2007). Nitric oxide affects preimplantation embryonic development in a rotating wall vessel bioreactor simulating microgravity. *Cell Biology International*.

[28] Ning L.-N., Lei X.-H., Cao Y.-J. (2015). Effect of short-term hypergravity treatment on mouse 2-cell embryo development. *Microgravity Science Technology*.

[29] Lei X., Cao Y., Zhang Y. (2018). Effect of microgravity on proliferation and differentiation of embryonic stem cells in an automated culturing system during the TZ-1 space mission. *Cell Proliferation*.

[30] Lei X., Cao Y., Zhang Y., Duan E., Duan E., Long M. Advances of mammalian reproduction and embryonic development under microgravity. *Life Science in Space: Experiments on Board the SJ-10 Recoverable Satellite*.

[31] Chen Q., Lin G., Wu N. (2016). Early exposure of rotating magnetic fields promotes central nervous regeneration in planarian Girardia sinensis. *Bioelectromagnetics*.

[32] Novikov V. V., Sheiman I. M., Fesenko E. E. (2008). Effect of weak static and low-frequency alternating magnetic fields on the fission and regeneration of the planarian Dugesia (Girardia) tigrina. *Bioelectromagnetics*.

[33] Gang N., Parker G. H., Lafrenie R. M., Persinger M. A. (2013). Intermittent exposures to nanoTesla range, 7 Hz, amplitude-modulated magnetic fields increase regeneration rates in planarian. *International Journal of Radiation Biology*.

[34] Van Huizen A. V., Morton J. M., Kinsey L. J. (2019). Weak magnetic fields alter stem cell-mediated growth. *Science Advances*.

[35] Goodman R., Lin-Ye A., Geddis M. S. (2009). Extremely low frequency electromagnetic fields activate the ERK cascade, increase hsp70 protein levels and promote regeneration in Planaria. *International Journal of Radiation Biology*.

[36] Murugan N. J., Karbowski L. M., Mekers W. F., Persinger M. A. (2015). Group planarian sudden mortality: is the threshold around global geomagnetic activity≥K6?. *Communicative & Integrative Biology*.

[37] Gang N., Persinger M. A. (2011). Planarian activity differences when maintained in water pre-treated with magnetic fields: a nonlinear effect. *Electromagnetic Biology and Medicine*.

[38] Lillywhite H. B. (1971). Temperature selection by the bullfrog, Rana catesbeiana. *Comparative Biochemistry and Physiology. A, Comparative Physiology*.

[39] Bennett S., Duarte C. M., Marbà N., Wernberg T. (2019). Integrating within-species variation in thermal physiology into climate change ecology. *Philosophical Transactions of the Royal Society of London. Series B, Biological Sciences*.

[40] Brattstrom B. H. (1968). Thermal acclimation in anuran amphibians as a function of latitude and altitude. *Comparative Biochemistry and Physiology*.

[41] Agathocleous M., Harris W. A. (2013). Metabolism in physiological cell proliferation and differentiation. *Trends in Cell Biology*.

[42] Folmes C. D. L., Terzic A. (2016). Energy metabolism in the acquisition and maintenance of stemness. *Seminars in Cell & Developmental Biology*.

[43] Salabei J. K., Lorkiewicz P. K., Holden C. R. (2015). Glutamine regulates cardiac progenitor cell metabolism and proliferation. *Stem Cells*.

[44] Gaspar J. A., Doss M. X., Hengstler J. G., Cadenas C., Hescheler J., Sachinidis A. (2014). Unique metabolic features of stem cells, cardiomyocytes, and their progenitors. *Circulation Research*.

[45] Gauron C., Rampon C., Bouzaffour M. (2013). Sustained production of ROS triggers compensatory proliferation and is required for regeneration to proceed. *Scientific Reports*.

[46] Love N. R., Chen Y., Ishibashi S. (2013). Amputation-induced reactive oxygen species are required for successful Xenopus tadpole tail regeneration. *Nature Cell Biology*.

[47] Osuma E. A., Riggs D. W., Gibb A. A., Hill B. G. (2018). High throughput measurement of metabolism in planarians reveals activation of glycolysis during regeneration. *Regeneration (Oxf)*.

[48] Pirotte N., Stevens A. S., Fraguas S. (2015). Reactive oxygen species in planarian regeneration: an upstream necessity for correct patterning and brain formation. *Oxidative Medicine and Cellular Longevity*.

[49] Doonan R., McElwee J. J., Matthijssens F. (2008). Against the oxidative damage theory of aging: superoxide dismutases protect against oxidative stress but have little or no effect on life span in Caenorhabditis elegans. *Genes & Development*.

[50] Andziak B., O'Connor T. P., Qi W. (2006). High oxidative damage levels in the longest-living rodent, the naked mole-rat. *Aging Cell*.

[51] Zhou W., Zhu M., Wilson M. A., Petsko G. A., Fink A. L. (2006). The oxidation state of DJ-1 regulates its chaperone activity toward *α*-Synuclein. *Journal of Molecular Biology*.

[52] Taira T., Saito Y., Niki T., Iguchi-Ariga S. M. M., Takahashi K., Ariga H. (2004). DJ-1 has a role in antioxidative stress to prevent cell death. *EMBO Reports*.

[53] Tsushima J., Nishimura K., Tashiro N. (2012). Protective effect of planarian DJ-1 against 6-hydroxydopamine-induced neurotoxicity. *Neuroscience Research*.

[54] Inoue T., Yamashita T., Agata K. (2014). Thermosensory signaling by TRPM is processed by brain serotonergic neurons to produce planarian thermotaxis. *The Journal of Neuroscience*.

[55] Ding X., Song L., Han Y. (2019). Effects of Fe3+ on Acute Toxicity and Regeneration of Planarian (Dugesia japonica) at Different Temperatures. *BioMed Research International*.

[56] Hammoudi N., Torre C., Ghigo E., Drancourt M. (2018). Temperature affects the biology of Schmidtea mediterranea. *Scientific Reports*.

[57] Best J. B., Goodman A. B., Pigon A. (1969). Fissioning in planarians: control by the brain. *Science*.

[58] Arnold C. P., Benham-Pyle B. W., Lange J. J., Wood C. J., Sánchez Alvarado A. (2019). Wnt and TGF*β* coordinate growth and patterning to regulate size-dependent behaviour. *Nature*.

[59] Herath S., Lobo D. (2020). Cross-inhibition of Turing patterns explains the self-organized regulatory mechanism of planarian fission. *Journal of Theoretical Biology*.

[60] Bueno D., Fernàndez-Rodríguez J., Cardona A., Hernàndez-Hernàndez V., Romero R. (2002). A novel invertebrate trophic factor related to invertebrate neurotrophins is involved in planarian body regional survival and asexual reproduction. *Developmental Biology*.

[61] Sakurai T., Lee H., Kashima M. (2012). The planarian P2X homolog in the regulation of asexual reproduction. *The International Journal of Developmental Biology*.

[62] Cole W. H. (1926). Temperature and locomotion in planaria. *The Journal of General Physiology*.

[63] de Oliveira M. S., Lopes K. A. R., Leite P. M. S. C. M., Morais F. V., de Campos Velho N. M. R. (2018). Physiological evaluation of the behavior and epidermis of freshwater planarians (Girardia tigrinaandGirardiasp.) exposed to stressors. *Biol Open*.

[64] Bowen I. D., Ryder T. A., Thompson J. A. (1974). The fine structure of the planarianPolycelis tenuis Iijima. *Protoplasma*.

[65] Smales L. R., Blankespoor H. D. (1978). The epidermis and sensory organs of Dugesia tigrina (Turbellaria: Tricladida). A scanning electron microscope study. *Cell and Tissue Research*.

[66] Brown H. M., Ogden T. E. (1968). The electrical response of the planarian ocellus. *The Journal of General Physiology*.

[67] Portner H. O. (2002). Climate variations and the physiological basis of temperature dependent biogeography: systemic to molecular hierarchy of thermal tolerance in animals. *Comparative Biochemistry and Physiology. Part A, Molecular & Integrative Physiology*.

[68] Finkel T. (2011). Signal transduction by reactive oxygen species. *The Journal of Cell Biology*.

[69] Abele D., Heise K., Pörtner H. O., Puntarulo S. (2002). Temperature-dependence of mitochondrial function and production of reactive oxygen species in the intertidal mud clam Mya arenaria. *The Journal of Experimental Biology*.

[70] Jarmuszkiewicz W., Woyda-Ploszczyca A., Koziel A., Majerczak J., Zoladz J. A. (2015). Temperature controls oxidative phosphorylation and reactive oxygen species production through uncoupling in rat skeletal muscle mitochondria. *Free Radical Biology & Medicine*.

[71] Gnaiger E. (2009). Capacity of oxidative phosphorylation in human skeletal muscle. *The International Journal of Biochemistry & Cell Biology*.

[72] Lemieux H., Blier P. U., Gnaiger E. (2017). Remodeling pathway control of mitochondrial respiratory capacity by temperature in mouse heart: electron flow through the Q-junction in permeabilized fibers. *Scientific Reports*.

[73] Lemieux H., Semsroth S., Antretter H., Höfer D., Gnaiger E. (2011). Mitochondrial respiratory control and early defects of oxidative phosphorylation in the failing human heart. *The International Journal of Biochemistry & Cell Biology*.

[74] Lemieux H., Warren B. E. (2012). An animal model to study human muscular diseases involving mitochondrial oxidative phosphorylation. *Journal of Bioenergetics and Biomembranes*.

[75] Scott K. Y., Matthew R., Woolcock J., Silva M., Lemieux H. (2019). Adjustments in the control of mitochondrial respiratory capacity to tolerate temperature fluctuations. *Journal Experimental Biology*.

[76] Gallio M., Ofstad T. A., Macpherson L. J., Wang J. W., Zuker C. S. (2011). The coding of temperature in the Drosophila brain. *Cell*.

[77] Arenas O. M., Zaharieva E. E., Para A., Vásquez-Doorman C., Petersen C. P., Gallio M. (2017). Activation of planarian TRPA1 by reactive oxygen species reveals a conserved mechanism for animal nociception. *Nature Neuroscience*.

[78] Ma K. X., Chen G. W., Liu D. Z. (2012). cDNA cloning of heat shock protein 90 gene and protein expression pattern in response to heavy metal exposure and thermal stress in planarian Dugesia japonica. *Molecular Biology Reports*.

[79] Dong Z., Cheng F., Yuwen Y. (2015). Identification and expression analysis of a Spsb gene in planarian Dugesia japonica. *Gene*.

[80] Hamana K., Hamana H., Shinozawa T. (1995). Alterations in polyamine levels of nematode, earthworm, leech and planarian during regeneration, temperature and osmotic stresses. *Comparative Biochemistry and Physiology. Part B, Biochemistry & Molecular Biology*.

[81] Carter J. A., Lind C. H., Truong M. P., Collins E.-M. S. (2015). To each his own. *Journal of Statistical Physics*.

[82] Hughes-Fulford M. (2004). Lessons learned about spaceflight and cell biology experiments. *Journal of Gravitational Physiology*.

[83] Warren P., Golden A., Hanover J., Love D., Shephard F., Szewczyk N. J. (2013). Evaluation of the fluids mixing enclosure system for life science experiments during a commercial Caenorhabditis elegans spaceflight experiment. *Advances in Space Research*.

[84] Vista SSEP Mission 11 Team, Hagstrom D., Bartee C., Collins E.-M. S. (2018). Studying planarian regeneration aboard the International Space Station within the Student Space Flight Experimental Program. *Frontiers in Astronomy and Space Sciences*.

